# Myocardial edema in acute myocarditis: relationship of T2 relaxometry and late enhancement burden by using dual-contrast turbo spin-echo MRI

**DOI:** 10.1007/s10554-017-1170-7

**Published:** 2017-05-20

**Authors:** A. Mayr, G. Klug, H. J. Feistritzer, S. J. Reinstadler, M. Reindl, R. Esterhammer, G. Feuchtner, B. Metzler, M. F. Schocke

**Affiliations:** 1 University Hospital for Radiology, Medical University of Innsbruck, Anichstrasse 35, 6020 Innsbruck, Austria; 2 University Hospital for Internal Medicine III (Cardiology and Angiology), Medical University of Innsbruck, Anichstrasse 35, 6020 Innsbruck, Austria

**Keywords:** Acute myocarditis, T2 relaxometry, TroponinT, Left ventricular function, Cardiac magnetic resonance

## Abstract

To quantify myocardial edema by using a T2 relaxometry approach with a dual-contrast turbo spin-echo (dcTSE) sequence in patients with acute myocarditis regarding focal late gadolinium enhancement (LGE) burden. CMR T2 relaxometry was performed in 39 patients (age 41 ± 19 years; 36% women) with LGE in a typical myocarditis pattern and in ten healthy volunteers (age 46 ± 12; 60% woman). dcTSE sequence (echo time 29 and 75 ms, respectively) was used for T2 mapping, analysis were performed on the basis of region of interest (ROI). Myocardial T2 relaxation times (T2 RT) in patients-ROI with focal LGE were significantly (p < 0.001) higher than T2 RT in patients—ROI without apparent LGE pattern (65 ms (IQR 36–95) vs. 60 ms (IQR 26–88), respectively). T2 RT in healthy volunteers [55 ms (IQR 35–71)] were significantly lower than in patients ROI with or without focal LGE-pattern (p < 0.001, respectively). T2 RT assessed by dcTSE are significantly higher in patients segments with and without focal LGE compared to normal controls, supporting a global myocardial inflammatory process in acute myocarditis. Furthermore, this quantitative T2-mapping approach highlights the potential to identify patients with diffuse myocarditis.

## Introduction

The diagnosis of myocarditis remains challenging in daily clinical routine due to its wide and varying range of symptoms in terms of patients clinical presentation. Acute inflammatory cardiomyopathies are responsible for 10% of sudden cardiac deaths in adults and further, are responsible for up to 40% of acute nonischemic cardiomyopathies [1, 2]. The diagnostic work-up of patients with suspected myocarditis is arranged via multiparametric approach including all available resources, i.e. cardiac biomarkers, advanced echocardiography, cardiac magnetic resonance (CMR) imaging and, in selected cases, endomyocardial biopsy. CMR plays a pivotal role in the diagnosis of acute myocarditis, offers important prognostic information and shows promise for clinical risk stratification [3, 4]. Using the recommended diagnostic criteria for active myocarditis (“Lake Louise Criteria”), encompassing the combination of edema-sensitive imaging, early and late gadolinium enhancement (LGE), sensitivities and specificities of ≈80% in recent-onset cases are achievable [4]. Edema-sensitive T2-weighted sequences can depict signal increases within affected myocardium but the extent of edema is difficult to quantify. However, quantitative myocardial T2 mapping has been recently shown to reliably identify myocardial involvement in patients with myocarditis [5, 6]. Our purpose was to further characterize myocardial segments with and without replacement LGE in a patient collective with symptomatic myocarditis and LGE-positive CMR by a T2 relaxometry approach using a dual-contrast turbo spin-echo (dcTSE) sequence. Furthermore, we investigated the relation between myocardial T2 relaxation times and biochemical markers of myocardial damage and ventricular dysfunction.

## Materials and methods

This retrospective study was approved by the ethics committee of Medical University of Innsbruck.

### Study population

We enrolled 39 patients, who were referred for clinical indications to CMR, into this study. Inclusion criteria were (1) symptoms and signs suggestive of cardiac disease (chest pain, dyspnea and palpitations), (2) evidence of myocardial injury as defined by elevated serum markers, such as creatine kinase (CK) or troponin T (TnT) and/or elevation of C-reactive protein (CRP) and (3) exclusion of coronary artery disease by angiography and / or clinical criteria and (4) apparent myocardial hyperenhancement by LGE in typical myocarditis pattern. Exclusion criteria were contraindications for CMR, hemodynamic instability, previous myocardial infarction and previous episode of myocarditis. Imaging scans were performed at a median of 4 days [interquartile range (IQR) 2–11] after symptom onset.

### Biochemical measurements

CK activity, TnT, N-terminal pro brain natriuretic peptide (NT-proBNP) as well as CRP values were drawn routinely at time of patients presentation in acute cardiac care as well as daily until discharge and determined by an enzymatic assay (Roche Diagnostics^®^, Mannheim, Germany). In each case, maximum values of these biochemical markers in acute stage of disease were included in statistical analysis.

### CMR protocol

All scans were performed on a 1.5 T Magnetom AVANTO-scanner (Siemens, Erlangen, Germany) using a 12-channel phased-array coil.

Cine imaging in 3 long axis planes (horizontal, vertical and 3 chamber) and short-axis planes covering the left ventricle (LV) were acquired using breath hold, retrospective ECG-triggered true Fast Imaging with Steady State Precession (trueFISP) bright-blood sequences. Evaluation of images was performed by an experienced observer using standard software (ARGUS, Siemens, Erlangen, Germany). Contouring of LV endo- and epicardial borders was performed semi-automatically. Papillary muscles were excluded from the myocardial mass and included to the LV volume. The most basal slices were excluded from evaluation in the presence of LV outflow tract, especially on the end systolic images [7].

**Fig. 1:**
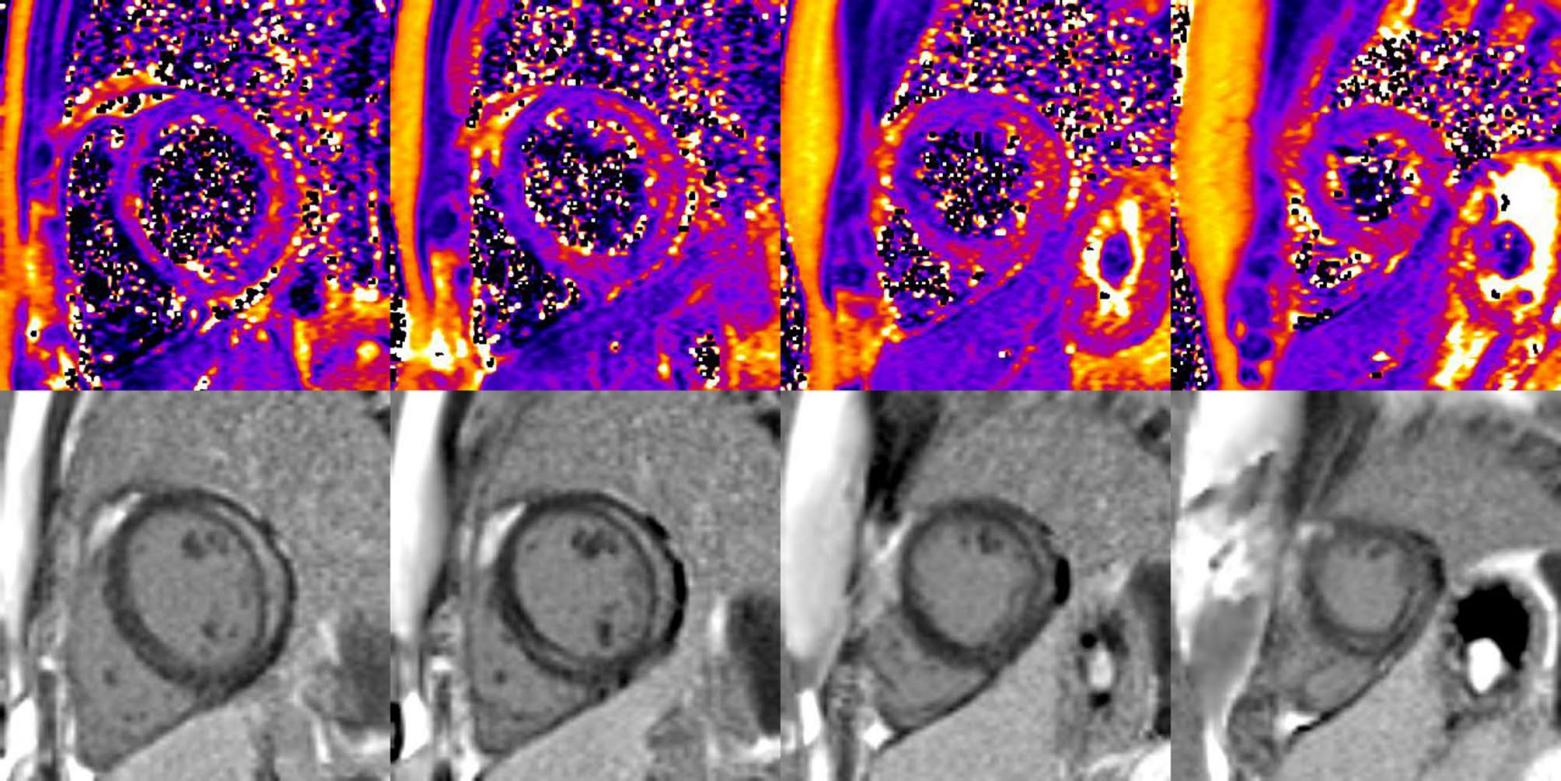
Exemplary set of T2 maps in basal, midventricular and apical slices of a patient with distinctive replacement LGE at subepicardial and midwall location, acquired with T2 dcTSE sequence

dcTSE dark blood sequence (echo times (TE) 29 and 75 ms, respectively; repetition time (TR) 762), field of view (FOV) 320 × 265, Trigger pulse 2, Matrix 256 × 159, slice thickness 8 mm, Echo-train length 9, generalized autocalibrating partially parallel acquisition (GRAPPA) factor 2 were acquired in short-axis slices covering the LV in an attempted breath hold. T2 maps were generated from these images by fitting a monoexponential decay curve at each pixel. (Fig. 1) T2 values were recorded from quantitative T2 maps for 16 LV segments by drawing regions of interest (ROI) encompassing each myocardial segment. Furthermore a ROI was placed into the shoulder muscles for normalization of the myocardial T2 values.

Ten minutes after a bolus injection of 0.1 mmol/kg gadolinium-based contrast agent (Multihance, Bracco, Vienna, Austria) with an infusion pump at 2–3 ml/s, we acquired LGE CMR images by using an ECG triggered phase sensitive inversion recovery (PSIR) single shot trueFISP sequence with consecutive short-axis slices as described in detail previously [8].

### Statistical analysis

We used *R 2.10.1* (*Team R Development Core (2009) R: A Language and Environment for Statistical Computing*) for statistical analysis. To test for normal distribution (ND) Shapiro–Wilk test was applied. Results for continuous variables are all expressed as mean ± standard deviation (SD) if they are ND or as median with corresponding IQR if not. To test for linear correlations of continuous variables Pearson's test (ND) or Spearman's rank correlations (not ND) were calculated.

Group differences were assessed by One-way ANOVA or the Kruskal–Wallis test, depending on data distribution, whereby significant effects were further evaluated posthoc testing with Bonferroni correction. Intra- and inter-observer variability was assessed via intra-class correlation coefficients (ICC). A two tailed p value <0.05 was considered to indicate statistical significance.

## Results

### Patient characteristics

Patient characteristics are summarized in Table 1. Thirty-nine patients (64% male, 36% female) were included. The mean age of the study cohort was 41 ± 19 years (range 14–77 years) and did not differ between male and female (p = 0.98). None of the patients had a history of cardiovascular disease. CMR was performed at a median of 4 days (IQR 2–11) after admission to the hospital. Patients LV systolic function overall was mildly reduced, with LV ejection fraction (LV-EF) averaging 51 ± 7.1%, end diastolic volume (EDV) and end systolic volume (ESV) reached 130 ± 43 and 65 ± 27 ml, respectively. The mean age of the volunteer cohort was 46 ± 12 years (range 24–61 years) and did not differ to patients age (p = 0.41), whereas mean LV-EF in healthy volunteers (61 ± 6%) was significantly higher than global LV function in patients group (p < 0.001).

**Table 1:** Patient's characteristics of the study cohort (n = 39)

**Variables**	
Age (years)	41 ± 19
Male sex [n (%)]	25 (64)
Body mass index [kg/m^2^]	23.5 ± 3.7
Heart rate (beats/min)	68 ± 8.5
CMR findings	
LV-EF (%)	51 ± 7.1
ESV (ml)	65 ± 27
EDV (ml)	130 ± 43
LGE mass [g (% of LVMM)]	8.2 ± 3.6 (8 ± 2.9%)
Troponin T (ng/l)	248 [15–2010]
C-reactive protein (mgdl)	3.46 [0.65–21.2]
Creatine kinase (U/L)	297 [192–1452]
NT-proBNP (ng/l)	741 [92–5638]

Data are presented as mean ± standard deviation, median plus interquartile range or frequencies plus percentage

*CMR* cardiac magnetic resonance, *LV-EF* left ventricular ejection fraction, *ESV* end-systolic volume, *EDV* end-diastolic volume, *LGE* late gadolinium enhancement, *LVMM* left ventricular myocardial mass

### Segment analysis of T2 relaxation times

**Fig. 2:**
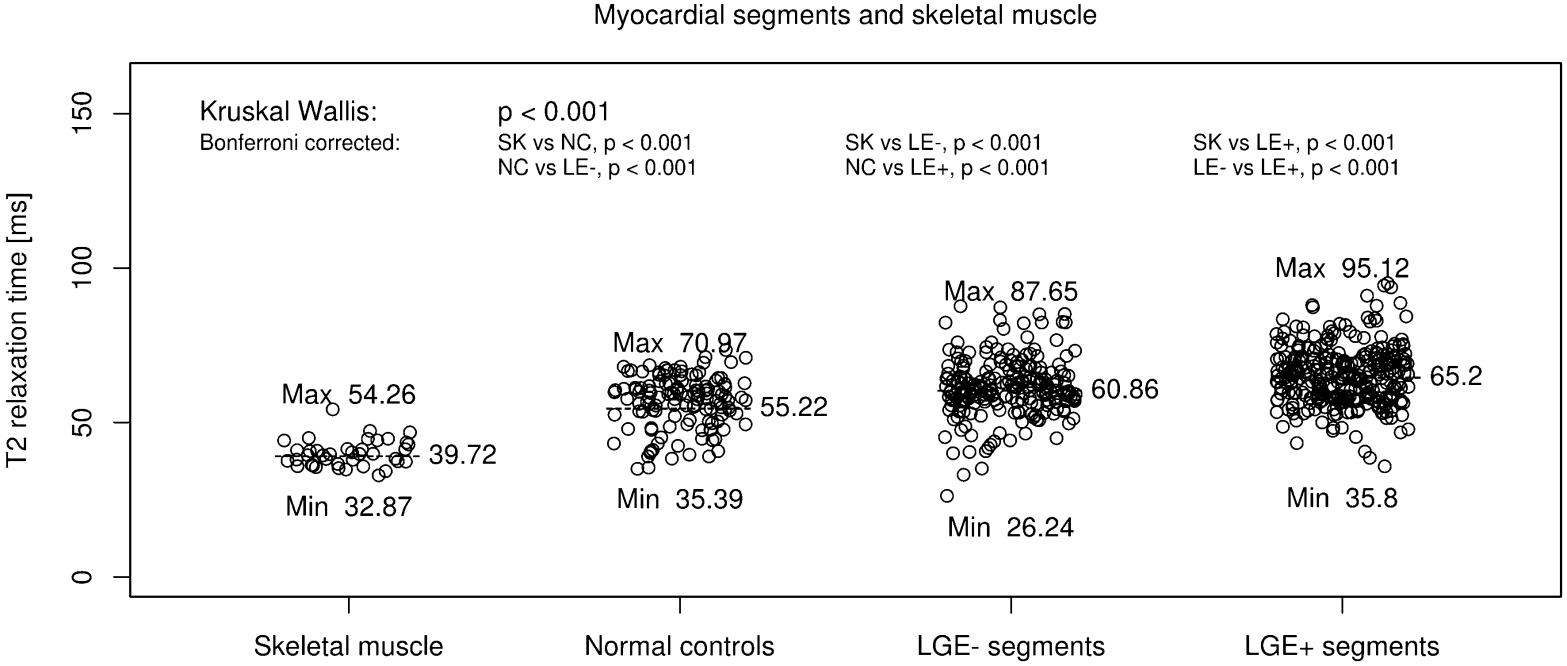
T2 relaxation times (T2 RT) in segments with focal replacement LGE (LGE-positive) was 65 ms (IQR 36–95) and were significantly higher (p < 0.001) than T2 RT in patients myocardial segments without LGE (LGE-negative) [60 ms (IQR 26–88)]. Myocardial T2 RT in normal controls [55 ms (IQR 35–71)] were significantly lower than T2 times in both, LGE-negative and LGE-positive segments (p < 0.001, respectively). ROI’s in skeletal muscle showed significantly lower T2 times [40 ms (IQR 33–54)] than in myocardial segments of normal controls or patients with and without LGE (p < 0.001, respectively)

Out of all analysed patients myocardial segments (624), 5.3% were not considerably due to qualitative impairment. Among the 591 finally included segments 26% (154) showed visible replacement LGE, whereas 437 (74%) did not. T2 relaxation times (T2 RT) in segments with replacement LGE was with 65 ms (IQR 36–95) significantly higher (p < 0.001) than T2 RT in patients myocardial segments without visually detectable LGE [60 ms (IQR 26–88)]. Myocardial T2 RT in healthy volunteers [55 ms (IQR 35–71)] were significantly lower than T2 times of patients segments with focal and more diffuse LGE (both p < 0.001). Figure 2 T2 RT in skeletal muscle ROI of patients and healthy volunteers did not show significant differences 40 ms (33–54 ms) and 39 ms (IQR 33–47), respectively (p = 0.97). We demonstrated excellent intra- and inter-observer agreement in T2 values (ICC for intra-observer agreement: 0.93 and ICC for inter-observer agreement: 0.96 for T2 relaxometry).

### T2 relaxation times and correlation with biochemical markers

Overall median T2 RT of patients was significantly correlated with maximum cTnT levels (r² = 0.319, p < 0.003) as well as with maximum CK levels (r² = 0.205, p < 0.05). T2 RT were not associated with maximum CRP (r² = 0.168, p = 0.836) and maximum NT-proBNP (r² = 0.036, p = 0.375) values.

### T2 relaxation times and correlation with cardiac function parameters

Inverse associations between T2 RT and CMR estimated parameters of LV function (LV-EF, EDV, ESV) were observed, but correlation was not significant.

## Discussion

In the present study we conducted a quantitative T2-mapping approach by using T2 weighted dcTSE sequence in dark blood preparation to identify a segmental based edematous myocardial involvement in patients with acute myocarditis and LGE—positive CMR. The main finding of this study are: (a) T2 RT in patients with LGE-positive myocarditis were significantly higher in ROI’s with focal LGE burden than in myocardial segments without LGE, (b) myocardial T2 RT in normal controls were significantly lower than in patients with myocarditis, both in segments with or without LGE burden, (c) overall mean myocardial T2 RT of myocarditis patients showed moderate but significant correlations to biomarkers of myocardial damage, (d) but neither to biomarkers of ventricular dysfunction (NT-proBNP), inflammatory markers (CRP) or myocardial function parameters.

Myocarditis, commonly caused by viral infections, is an underdiagnosed cause of acute heart failure, sudden death and chronic dilated cardiomyopathy [9]. The clinical manifestations of acute myocarditis vary widely from asymptomatic to seriously ill. The definite diagnosis can be obtained by biopsy, whereby cardiac MRI is emerging as an important tool for the diagnosis and follow-up of patients and for guidance of endomyocardial biopsy [10]. The MRI criteria for the diagnosis of myocarditis mainly comprise LGE and functional impairment. Problems occur, when the LGE is not associated by a restriction of LV function or the LGE is rather diffuse and not well delineated. The quantitative measurement of LGE within the myocardium remains very difficult. Some previous studies could show signal increases on T2 weighted images with double or triple inversion recovery [3, 11]. Therefore, dark blood T2-weighted imaging is the recommended sequence for the detection of myocardial edema [4]. However, several challenges and limitations of T2 weighted techniques have emerged, especially when employing a T2 short tau inversion-recovery sequence. Presence of arrhythmia as well as poor breath holding with subsequent respiratory artifacts may degrade image quality. Moreover nulling of the LV blood pool signal may be imperfect in subendocardial areas with slow LV cavity blood flow and impairs the image contrast in these areas due to bright signal artifacts [12]. Even though some of these limitations were found to be reduced in newer bright-blood T2-weighted sequences, the evaluation of global myocardial edema and myocarditis remains superior by using db T2-weighted imaging [13, 14]. The quantification of the T2 signal is a well established method for tissue characterization, and provide an objective quantitative parameter with superiority over subjective image reading [15]. Since the increased free myocardial water content in myocardial inflammation prolongs T2 and T1 relaxation times, myocardial T1 and T2 mapping techniques have been shown to pixel-wise quantify myocardial water and offer superior diagnostic performance as compared to conventional T2-weighted techniques in terms of image- based visual detection of edema [5, 16].

Accordingly, our patient collective exhibited increased T2 RT within all myocardial segments compared to normal controls, even within segments with no visible LGE. Consecutively, this finding highlights the potential of T2 relaxometry approaches to identify inflammatory affected myocardium even in the absence of late enhancement.

Furthermore, our main finding confirms that the inflammatory process affects the whole myocardium and is also supported by a recent study investigating T2 and T1 mapping as well as extracellular volume (ECV) in patients with myocarditis, whereby the mapping methods were superior to the visual inspection [17]. T2 mapping appears to be a reliable tool for the detection of both focal and diffuse myocardial alterations. However, the substantial overlap in T2 between the different groups might by caused by our segment-specific approach. Commonly, the inflammatory process affects primary the epicardial site of the myocardium. However, it might be rare that the whole thickness of a myocardial segment is homogeneously infested by the inflammatory process which consequently could explain a diminution of group differences due partial volume effects.

Our results rely mainly on T2 mapping derived from the dcTSE sequence with dark blood preparation. This sequence provides diagnostic relevant images with the additional feature to calculate T2 RT [18]. Recent studies reported that T1 mapping is capable for depicting tissue changes in acute myocarditis such as edema, hyperemia, and necrosis or scarring, with a higher sensitivity for the detection of myocarditis compared to T2 mapping and LGE [19–22]. Nevertheless, T2 mapping is a well established method for quantification of tissue edema, while T1 mapping is a relatively new method for the assessment of myocardial edema. Another advantage of the quantitative T2 mapping approach utilized in our study is its easy implementation into a clinical MR protocol on nearly each commercially available MR scanner. In contrast, T1 mapping is technically only available on a limited number of MR units.

In our patient cohort, we observed a moderate correlation between T2 RT and maximum deflection of cTnT as well as CK in the acute stage of disease. Consequently, increased T2 relaxation might be an adequate marker for the determination of myocardial affection reflecting biochemical severity of myocarditis.

However, T2 relaxation failed to correlate with CRP, NT-proBNP and LV function. CRP as a marker of inflammatory processes is known to play a central role in the pathogenesis of atherosclerosis and its elevated plasma levels have been found to predict future cardiovascular risk [23, 24]. However, in line with our results Mewton et al. failed to show correlations of CRP with conditions of myocardial inflammation [25]. NT-pro BNP is released in response to increased LV wall stretch and elevated NT-proBNP levels correlate with severe LV dysfunction [26]. Missing correlations of NT-proBNP as well as left-ventricular function parameters with T2 relaxation in our study could be linked with the relatively low functional impairment of our study group.

### Limitations

The study has some important limitations. First, our study comprises only data from patients with symptomatic myocarditis and focal replacement LGE in at least one of the myocardial segments. Our data however, support a widespread global myocardial inflammatory process of the whole heart, even of the LGE-negative segments in the acute stage of myocarditis. Since imaging scans were performed at a median of 4 days after symptom onset, the highlighted potential of this quantitative T2-mapping approach to identify patients with diffuse myocarditis is limited to the acute stage of the disease. The presented data can not give any statement about T2 mapping in monitoring advanced disease stages. However, recently von Knobelsdorff-Brenkenhoff et al. in a multiparametric CMR mapping approach emphasized only T2 mapping to discriminate between acute and healed stages of myocarditis [27].

Furthermore, the presented correlations of T2 relaxation times with biochemical markers are univariate correlations.

The discussion is partly based on the speculation that LGE-negative segments in our patient collective are comparable to myocarditis patient without any replacement LGE.

Furthermore, this is a single-center investigation with a small sample size and therefore confirmation in larger studies would be important.

## Conclusions

In conclusion, T2 relaxometry shows significant differences between myocardial segments with and without LGE burden in patients with acute myocarditis, highlighting its potential to indicate inflammatory processes without any replacement LGE. Therefore, our results might suggest that T2 values provide additional information about the widespread global myocardial inflammatory process in the acute stage of myocarditis, which is related to the biochemical severity of myocarditis.
